# Correction: Prevalence of peripheral arterial disease and arterial calcification based on three ankle-brachial index calculation methods (highest, average, and lowest systolic ankle pressure): A cross-sectional study in Type 2 diabetes mellitus patients in Peru

**DOI:** 10.1371/journal.pone.0344591

**Published:** 2026-03-09

**Authors:** The PLOS One Staff

The fifth author, Manolo Briceño-Alvarado, is incorrectly noted as the corresponding author. The correct corresponding author is Marlon Yovera-Aldana. Marlon Yovera-Aldana’s email address is: myovera@cientifica.edu.pe.

The publisher apologizes for the error.
