# Supplementary material for: Prevalence of peripheral arterial disease and arterial calcification based on three ankle-brachial index calculation methods (highest, average, and lowest systolic ankle pressure): A cross-sectional study in Type 2 diabetes mellitus patients in Peru
Source: PLoS One. 2025 Sep 18;20(9):e0316981. doi: 10.1371/journal.pone.0316981 (PMC12445549; doi:10.1371/journal.pone.0316981)
Supplement: S1 Table — (DOCX) [file pone.0316981.s001.docx]

S1 Table. Comparison of baseline characteristics between included and non-included patients

| Variable | Included | Excluded | p-value |
| --- | --- | --- | --- |
| Demographics |  |  |  |
| Sex (Female) | 69.8% | 68.6% | 0.685 |
| Age, mean (SD) | 61.4 (11.0) | 59.3 (11.0) | 0.002 |
| Education |  |  |  |
| None (%) | 6.2% | 4.8% | 0.516 |
| Elementary (%) | 34.1% | 35.4% |  |
| High School (%) | 48.5% | 50.8% |  |
| College (%) | 11.2% | 9.0% |  |
| Past medical history |  |  |  |
| Diabetes duration, mean (SD) | 8.88 (7.55) | 8.69 (7.67) | 0.704 |
| Hypertension (%) | 33.2% | 19.0% | <0.001 |
| Diabetes treatment |  |  |  |
| Diet only (%) | 5.3% | 3.7% | 0.027 |
| Oral agent only (%) | 56.8% | 63.0% |  |
| Oral agents + insulin (%) | 5.8% | 8.2% |  |
| Insulin only (%) | 32.2% | 25.0% |  |
| Clinica exam |  |  |  |
| BMI, mean (SD) | 27.73 (4.88) | 27.86 (4.94) | 0.772 |
| Neuropathy (%) | 81.3% | 71.8% | 0.001 |
| Laboratory findings |  |  |  |
| Glucose, mean (SD) | 172.9 (80.1) | 189.0 (87.3) | 0.024 |
| HbA1c, mean (SD) | 9.94 (3.74) | 10.53 (4.11) | 0.118 |
| LDL-c, mean (SD) | 120.5 (43.1) | 120.5 (47.5) | 0.981 |
| SBP, mean (SD) | 131.0 (19.8) | 125.6 (20.4) | 0.146 |
| eGFR, mean (SD) | 85.1 (27.4) | 88.5 (27.1) | 0.162 |
| Albuminuria, mean (SD) | 28.9 (122.4) | 23.9 (82.8) | 0.670 |
| Triglycerides, mean (SD) | 168.9 (95.0) | 183.7 (118.5) | 0.111 |

**SD. Standard deviation**
