# Supplementary material for: Prevalence of peripheral arterial disease and arterial calcification based on three ankle-brachial index calculation methods (highest, average, and lowest systolic ankle pressure): A cross-sectional study in Type 2 diabetes mellitus patients in Peru
Source: PLoS One. 2025 Sep 18;20(9):e0316981. doi: 10.1371/journal.pone.0316981 (PMC12445549; doi:10.1371/journal.pone.0316981)
Supplement: S2 Table — (DOCX) [file pone.0316981.s002.docx]

**S2 Table. Prevalence of PAD and AC According to the Highest SAP Criterion, by Clinical–Demographic Characteristics.**

|  | PAD  N (%) | Normal  N (%) | AC  N (%) |
| --- | --- | --- | --- |
| **Full sample** | 50 (7.8) | 476 (74.0) | 117 (18.2) |
| **Demographics** |  |  |  |
| **Sex** |  |  |  |
| Male | 16 (8.3) | 141 (72.7) | 37 (19.1) |
| Female | 34 (7.6) | 335 (74.6) | 80 (17.8) |
| **Age (years)** |  |  |  |
| < 75.0 | 39 (6.9 ) | 423 (74.5 ) | 106 (18.7) |
| ≥ 75.0 | 11 (14.5) | 53 (69.7) | 12 (15.8) |
| **Education level** |  |  |  |
| Elementary or minus | 27 (10.5 ) | 192 (74.4 ) | 39 (15.1) |
| High-School or more | 23 (6.0 ) | 284 (73.8) | 78 (20.3) |
| **Past medical history** |  |  |  |
| **Duration of diabetes (years)** |  |  |  |
| < 10.0 | 21 (5.5) | 300 (78.5) | 61 (16.0) |
| 10 to 19.9 | 16 (8.9) | 135 (75.0) | 29 (16.1) |
| $\geq$20.0 | 13 (16.1) | 41 (50.6) | 27 (33.3) |
| **Diabetes treatment** |  |  |  |
| Oral agentes or not medication ^a^ | 31 (7.7) | 299 (74.6) | 71 (17.7) |
| Insulin with or without oral agents | 19 (7.9) | 177 (73.1) | 46 (19.0) |
| **Previous diabetic foot ulcer** |  |  |  |
| No | 40 (6.8) | 449 (76.4) | 99 (16.8) |
| Yes | 10 (18.2) | 27 (49.1) | 18 (32.7) |
| **Hypertension** |  |  |  |
| No | 18 (6.6) | 209 (76.8) | 45 (16.5) |
| Yes | 17 (12.5) | 96 (70.6) | 23 (16.9) |
| **Clinical evaluation** |  |  |  |
| **Peripheral neuropathy^b^** |  |  |  |
| No | 29 (6.4) | 343 (75.1) | 85 (18.6) |
| Yes | 21 (11.3) | 133 (71.5) | 32 (17.2) |
| **Altered foot pulses ^c^** |  |  |  |
| No | 24 (4.9) | 368 (75.4) | 96 (19.7) |
| Yes | 26 (16.8) | 108 (69.7) | 21 (13.6) |
| **BMI (**kg/m^2^**)** |  |  |  |
| < 25.0 | 20 (15.9) | 89 (70.6) | 17 (13.5) |
| $\geq$ 25.0 | 15 ( 5.0) | 216 (72.2) | 68 (22.7) |
| **Laboratory findings** |  |  |  |
| **eGFR<60 mL/min/1.73 m² ^d^** |  |  |  |
| No | 21 (6.7) | 232 (74.4) | 59 (18.9) |
| Yes | 10 (14.9) | 43 (64.2) | 14 (20.9) |

PAD: Peripheral arterial disease. ABI: Ankle-brachial index. BMI: Body mass. eGFR :Estimated glomerular filtration rate Index SAP: Systolic ankle pressure

^a^ Oral agents; (Metformin or glibenclamide). ^b^ Michigan Neuropathy Screening Instrument score >2. ^c^. Positive if there is an absence of pulse in any of the arteries: right or left pedal. right or left posterior tibial. ^d^. Estimated glomerular filtration rate calculated by the CKD-EPI.
