# Supplementary material for: Prevalence of peripheral arterial disease and arterial calcification based on three ankle-brachial index calculation methods (highest, average, and lowest systolic ankle pressure): A cross-sectional study in Type 2 diabetes mellitus patients in Peru
Source: PLoS One. 2025 Sep 18;20(9):e0316981. doi: 10.1371/journal.pone.0316981 (PMC12445549; doi:10.1371/journal.pone.0316981)
Supplement: S3 Table — (DOCX) [file pone.0316981.s003.docx]

**S3 Table. Prevalence of PAD and AC According to the Lowest SAP Criterion. by Clinical–Demographic Characteristics.**

|  | PAD  N (%) | Normal  N (%) | Calcificación  N (%) |
| --- | --- | --- | --- |
| Full sample | 181 (28.2) | 358 (55.7) | 104 (16.2) |
| **Demographics** |  |  |  |
| **Sex** |  |  |  |
| Male | 56 (28.9) | 105 (54.1) | 34 (17.0) |
| Female | 125 (27.8) | 253 (56.4) | 71 (15.8) |
| **Age (years)** |  |  |  |
| < 60.0 | 61 (21.7) | 161 (57.3) | 59 (21.0) |
| 60.0 to 74.9 | 91 (31.8) | 162 (56.6) | 33 (11.5) |
| ≥ 75.0 | 29 (38.2) | 35 (46.1) | 12 (15.8) |
| **Education level** |  |  |  |
| Elementary or minus | 84 (32.6) | 139 (53.9) | 35 (13.6) |
| High-School or more | 97 (25.2) | 219 (56.9) | 69 (17.9) |
| **Past medical history** |  |  |  |
| **Duration of diabetes (years)** |  |  |  |
| < 10.0 | 93 (24.4) | 236 (61.8) | 53 (13.9) |
| 10 to 19.9 | 58 (32.2) | 96 (53.3) | 26 (14.4) |
| $\geq$20.0 | 30 (37.0) | 26 (32.1) | 25 (30.9) |
| **Diabetes treatment** |  |  |  |
| Oral agentes or not medication ^a^ | 96 (23.9) | 241 (60.1) | 64 (16.0) |
| Insulin with/without oral agents | 85 (35.1) | 117 (48.3) | 40 (16.5) |
| **Previous diabetic foot ulcer** |  |  |  |
| No | 164 (27.9) | 329 (55.9) | 95 (16.2) |
| Yes | 21 (38.2) | 19 (34.5) | 15 (27.3) |
| **Hypertension** |  |  |  |
| No | 70 (25.7) | 162 (59.6) | 40 (14.7) |
| Yes | 45 (33.1) | 70 (51.5) | 21 (15.4) |
| **Clinical evaluation** |  |  |  |
| **Peripheral neuropathy^b^** |  |  |  |
| No | 118 (25.8) | 264 (57.8) | 75 (16.4) |
| Yes | 63 (33.9) | 94 (50.5) | 29 (15.6) |
| **Altered foot pulses ^c^** |  |  |  |
| No | 89 (18.2) | 312 (63.9) | 87 (17.8) |
| Yes | 92 (59.4) | 46 (29.7) | 17 (11.0) |
| **BMI (**kg/m^2^**)** |  |  |  |
| < 25.0 | 44 (34.9) | 68 (54.0) | 14 (11.1) |
| 25.0 to 29.9 | 54 (30.3) | 92 (51.7) | 32 (18.0) |
| $\geq$30.0 | 23 (19.0) | 69 (57.0) | 29 (24.0) |
| **Laboratory findings** |  |  |  |
| **eGFR<60 mL/min/1.73 m² ^d^** |  |  |  |
| No | 79 (25.3) | 181 (58.0) | 52 (16.7) |
| Yes | 30 (44.8) | 25 (37.3) | 12 (17.9) |

PAD: Peripheral arterial disease. ABI: Ankle-brachial index. BMI: Body mass. eGFR :Estimated glomerular filtration rate Index SAP: Systolic ankle pressure

^a^ Oral agents; (Metformin or glibenclamide). ^b^ Michigan Neuropathy Screening Instrument score >2. ^c^. Positive if there is an absence of pulse in any of the arteries: right or left pedal, right or left posterior tibial. ^d^ Estimated glomerular filtration rate calculated by the CKD-EPI.
