# Supplementary material for: Prevalence of peripheral arterial disease and arterial calcification based on three ankle-brachial index calculation methods (highest, average, and lowest systolic ankle pressure): A cross-sectional study in Type 2 diabetes mellitus patients in Peru
Source: PLoS One. 2025 Sep 18;20(9):e0316981. doi: 10.1371/journal.pone.0316981 (PMC12445549; doi:10.1371/journal.pone.0316981)
Supplement: S4 Table — (DOCX) [file pone.0316981.s004.docx]

**S4 Table. Prevalence of PAD and AC According to the Average SAP Criterion, by Clinical–Demographic Characteristics.**

|  | PAD  N (%) | Normal  N (%) | AC  N (%) |
| --- | --- | --- | --- |
| **Full sample** | 99 (15.4) | 473 (73.6) | 71 (11.0) |
| **Demographics** |  |  |  |
| **Sex** |  |  |  |
| Male | 28 (14.4) | 143 (73.7) | 23 (11.9) |
| Female | 71 (15.8) | 330 (73.5) | 48 (10.7) |
| **Age (years)** |  |  |  |
| < 60.0 | 22 (7.7) | 218 (77.6) | 41 (14.6) |
| ≥ 60.0 | 77 (21.3 ) | 255 (70.4) | 30 (8.3) |
| **Education level** |  |  |  |
| Elementary or minus | 46 (17.8) | 191 (74.0) | 21 (8.1) |
| High-School or more | 53 (13.8) | 282 (73.2) | 50 (13.0) |
| **Past medical history** |  |  |  |
| **Duration of diabetes (years)** |  |  |  |
| < 10.0 | 47 (12.3) | 297 (77.7) | 38 (9.9) |
| 10 to 19.9 | 31 (17.2) | 128 (71.1) | 21 (11.7) |
| $\geq$20.0 | 21 (25.9) | 48 (59.2) | 12 (14.8) |
| **Diabetes treatment** |  |  |  |
| Oral agentes or not medication ^a^ | 54 (13.0) | 296 ( 74.4) | 48 (12.1) |
| Insulin with or without oral agents | 45 (18.6) | 174 (71.9) | 23 (9.5) |
| **Previous diabetic foot ulcer** |  |  |  |
| No | 87 (14.8) | 439 (74.7) | 62 (10.5) |
| Yes | 12 (21.8) | 34 (61.8) | 9 (16.4) |
| **Hypertension** |  |  |  |
| No | 33 (12.1) | 214 (78.7) | 25 (9.2) |
| Yes | 31 (22.8) | 87 (64.0) | 18 (13.2) |
| **Clinical evaluation** |  |  |  |
| **Peripheral neuropathy^b^** |  |  |  |
| No | 63 (13.8) | 344 (75.3) | 50 (10.9) |
| Yes | 36 (19.4) | 129 (69.4) | 21 (11.3) |
| **Altered foot pulses ^c^** |  |  |  |
| No | 45 (9.2) | 385 (78.9) | 58 (11.9) |
| Yes | 54 (34.8) | 88 (56.8) | 13 (8.4) |
| **BMI (**kg/m^2^**)** |  |  |  |
| 25.0 to 29.9 | 26 (14.6) | 129 (72.5) | 23 (12.9) |
| $\geq$30.0 | 11 (9.1) | 85 (70.2) | 25 (20.7) |
| **Laboratory findings** |  |  |  |
| **eGFR<60 mL/min/1.73 m² ^d^** |  |  |  |
| No | 40 (12.8) | 233 (74.7) | 39 (12.5) |
| Yes | 16 (23.9) | 41 (61.2) | 10 (14.9) |

PAD: Peripheral arterial disease. ABI: Ankle-brachial index. BMI: Body mass. eGFR :Estimated glomerular filtration rate Index SAP: Systolic ankle pressure

^a^ Oral agents; (Metformin or glibenclamide). ^b^ Michigan Neuropathy Screening Instrument score >2. ^c^. Positive if there is an absence of pulse in any of the arteries: right or left pedal. right or left posterior tibial. ^d^. Estimated glomerular filtration rate calculated by the CKD-EPI.
