# Supplementary material for: Prevalence of peripheral arterial disease and arterial calcification based on three ankle-brachial index calculation methods (highest, average, and lowest systolic ankle pressure): A cross-sectional study in Type 2 diabetes mellitus patients in Peru
Source: PLoS One. 2025 Sep 18;20(9):e0316981. doi: 10.1371/journal.pone.0316981 (PMC12445549; doi:10.1371/journal.pone.0316981)
Supplement: S5 Table — (DOCX) [file pone.0316981.s005.docx]

**S5 Table. Demographic and clinical numerical variables of patients with diabetes mellitus**

|  | N | Peripheral arterial disease | Normal | Arterial calicification |
| --- | --- | --- | --- | --- |
| **Highest SAP** |  |  |  |  |
| Age; (Mean $\pm$ SD) | 643 | 68.8 $\pm$ 8.4 | 61,2 $\pm$11,0 | 59,3 $\pm$ 10,6 |
| Diabetes disease time; Median [IQR] | 643 | 11.5 [5 to 20] | 6 [ 3 to 12] | 8 [3 to 18] |
| BMI ^b^ Median [IQR] | 425 | 26.2 [23.8 to 29.1] | 27.9 [24.3 a 30.8] | 27.8 [25.7 a 33.3] |
| ABI; Median [IQR] | 325 | 8.9 [7.1 to 12.2] | 9.2 [7 to 11.5] | 9.3 [7.8 to 11.4] |
| LDL-cholesterol; Median [IQR] | 373 | 122 [87 to 154] | 119 [87 to 149] | 125 [96 to 152] |
| **Lowest SAP** |  |  |  |  |
| Age; (Mean $\pm$ SD) | 643 | 63.5 $\pm$11.4 | 60.9 $\pm$10.6 | 59,3 $\pm$ 11,1 |
| Diabetes disease time; Mediana [IQR] | 643 | 8 [4 to 15] | 5 [ 3 to 12] | 8 [4 to 18] |
| BMI ^b^ Median [IQR] | 425 | 26.2 [23.8 to 29.1] | 27.6 [24.3 to 30.8] | 27.8 [25.7 to 33.3] |
| ABI; Median [IQR] | 325 | 8.9 [7.1 to 12.2] | 9,2 [7 to 11.5] | 9.3 [7.8 to 11.4] |
| LDL-cholesterol; Median [IQR] | 373 | 122 [87 to 154] | 119 [87 to 149] | 125 [96 to 152] |
| **Average SAP** |  |  |  |  |
| Age; (Mean $\pm$ SD) | 643 | 65.6 $\pm$ 10.5 | 60,8 $\pm$10,9 | 59,0 $\pm$ 10,9 |
| Diabetes disease time; Mediana [IQR] | 643 | 10 [4 to 18] | 6 [ 3 to 12] | 8 [4 to 15] |
| BMI ^b^ Median [IQR] | 425 | 25.2 [23.3 to 28] | 27,6 [24.3 to 30.4] | 29.1 [26.3 to 33.5] |
| ABI; Median [IQR] | 325 | 8.9 [7.4 to 12.2] | 9.2 [6.9 to 11.5] | 9.5 [8 to 12.4] |
| LDL-cholesterol; Median [IQR] | 373 | 111 [83 to 156] | 122 [88 to 152] | 125 [93 to 143] |

ABI: ankle-Brachial Index, IQR: Interquartilic rank, BMI: Body mass index. SAP: Systolic ankle pressure
