# Supplementary material for: Prevalence of peripheral arterial disease and arterial calcification based on three ankle-brachial index calculation methods (highest, average, and lowest systolic ankle pressure): A cross-sectional study in Type 2 diabetes mellitus patients in Peru
Source: PLoS One. 2025 Sep 18;20(9):e0316981. doi: 10.1371/journal.pone.0316981 (PMC12445549; doi:10.1371/journal.pone.0316981)
Supplement: Attachment — Submitted filename: Response_to_Reviewers_English_PLOS_ONE.docx [file pone.0316981.s007.docx]

# Response to Reviewers – Manuscript PONE-D-24-58827

Manuscript Title: “Prevalence of Peripheral Arterial Disease and Arterial Calcification Based on Three Ankle–Brachial Index Calculation Methods (Highest, Average, and Lowest Systolic Ankle Pressure): A Cross-Sectional Study in Type 2 Diabetes Mellitus Patients in Peru”

Manuscript ID: PONE-D-24-58827

## Editorial Requirements

1. Observation: Please ensure that your manuscript meets PLOS ONE's style requirements, including those for file naming.

https://journals.plos.org/plosone/s/file?id=wjVg/PLOSOne_formatting_sample_main_body.pdf and 
https://journals.plos.org/plosone/s/file?id=ba62/PLOSOne_formatting_sample_title_authors_affiliations.pdf

Response: We have revised and formatted the manuscript and file names to meet PLOS ONE’s editorial requirements, following the provided templates.

2. Observation : Please provide additional details regarding participant consent. In the ethics statement in the Methods and online submission information, please ensure that you have specified (1) whether consent was informed and (2) what type you obtained (for instance, written or verbal, and if verbal, how it was documented and witnessed). If your study included minors, state whether you obtained consent from parents or guardians. If the need for consent was waived by the ethics committee, please include this information.

If you are reporting a retrospective study of medical records or archived samples, please ensure that you have discussed whether all data were fully anonymized before you accessed them and/or whether the IRB or ethics committee waived the requirement for informed consent. If patients provided informed written consent to have data from their medical records used in research, please include this information

Once you have amended this/these statement(s) in the Methods section of the manuscript, please add the same text to the “Ethics Statement” field of the submission form (via “Edit Submission”).

For additional information about PLOS ONE ethical requirements for human subjects research, please refer to http://journals.plos.org/plosone/s/submission-guidelines#loc-human-subjects-research..

Response: We have updated the Methods section and the submission form with the following statement:

Before: “Approval was obtained from the Institutional Ethics and Research Committee of Hospital María Auxiliadora under code HMA/CIEI/015/2024. The data did not contain any personally identifiable information.”

After: "This was a retrospective secondary analysis of anonymized medical records. Approval was obtained from the Institutional Ethics and Research Committee of Hospital María Auxiliadora (code HMA/CIEI/015/2024). Informed consent was waived by the committee, as the data were fully anonymized and no identifiable patient information was used or accessed at any point in the study."

## Reviewer #1 Comments and Responses

3. Observation: Page 1, Lines 1-4: The title indicates a cross-sectional study by mentioning "prevalence," but it does not explicitly state the study design (e.g., "A Cross-Sectional Study"). This lack of clarity may obscure the design for readers unfamiliar with prevalence as a hallmark of cross-sectional research.

Page 1, Lines 1-4: The title refers to "Different Methods of Ankle-Brachial Index Calculation" but fails to specify these methods (e.g., highest, lowest, average systolic pressure).

Response: The title has been revised to include the design and ABI methods calculation:

Before: “Different Methods of Ankle-Brachial Index Calculation on the Prevalence of Peripheral Arterial Disease and Arterial Calcification in Subjects with Type 2 Diabetes Mellitus from a Public Hospital in Peru ”

After: “Prevalence of Peripheral Arterial Disease and Arterial Calcification Based on Three Ankle-Brachial Index Calculation Methods (Highest, Average, and Lowest Systolic Ankle Pressure): A cross-sectional study in Type 2 Diabetes Mellitus Patients in Peru”

4. Observation: Page 1, Lines 25-48: The abstract omits a background statement, leaving the context and significance of studying PAD and AC in diabetic patients unclear.
   Page 1, Lines 29-36: The methods section vaguely describes ABI calculation ("using the lowest, highest, or average systolic pressure") without explaining the numerator and denominator or the measurement process.
   Page 1, Lines 37-43: Prevalence rates for PAD and AC are reported, but confidence intervals are not included in the abstract despite being present in the main text (e.g., Page 20, Lines 244-245).
   Page 1, Lines 44-48: The conclusions state a prevalence range but misreport "CA" instead of "AC" (likely a typographical error), introducing inconsistency. Additionally, the implications of the findings for clinical practice or research are not articulated beyond a call for further study.

Response:

We thank the reviewer for these detailed comments regarding the abstract. In response, we have revised the abstract to include a concise background statement highlighting the clinical relevance of studying peripheral arterial disease (PAD) and arterial calcification (AC) in patients with diabetes, particularly considering the heterogeneity introduced by different ankle-brachial index (ABI) calculation methods.

The methods section of the abstract now explicitly defines how the ABI was calculated, specifying the use of the highest brachial systolic pressure as the denominator, and the highest, lowest, or average systolic ankle pressures (from either dorsalis pedis or posterior tibial arteries) as the numerator. We also clarified that these methods were compared in a population of Peruvian adults with type 2 diabetes.

To improve the precision of reporting, we added 95% confidence intervals for the prevalence estimates of PAD and AC across the three ABI calculation methods. The typographical error "CA" has been corrected to "AC" to maintain consistency.

Finally, the conclusions have been expanded to better reflect the clinical and research implications of the findings, emphasizing the impact of ABI calculation methods on disease classification and the importance of method selection in clinical and epidemiological settings.

5. Page 10, Lines 52-100: The research question—determining PAD and AC prevalence using different ABI methods—is implied but not explicitly defined.

We thank the reviewer for this valuable observation. In response, we have revised the final paragraph of the Introduction to explicitly define the research question. The paragraph now reads: “Therefore, the objective of this study was to determine how the prevalence of peripheral arterial disease (PAD) and arterial calcification (AC) varies according to the method used to calculate the ankle-brachial index (ABI)—specifically, using the highest, average, or lowest ankle systolic pressure—in patients with type 2 diabetes mellitus at Hospital María Auxiliadora, a public hospital in Peru.”
This clarification ensures that the research question is explicitly stated and fully aligned with the study title, methods, and analytical framework.

Therefore, this study aims to determine how the prevalence of peripheral arterial disease and arterial calcification varies according to the method used to calculated the ankle-brachial index (highest, average or lowest ankle systolic pressure) in patients with type 2 diabetes mellitus at a public hospital in Peru..

6. Page 11, Lines 85-89: The rationale for using three ABI methods is mentioned (e.g., detecting less severe cases or assessing severity), but it lacks justification tied to prior evidence or clinical relevance specific to diabetic patients in Peru.

Response: We have incorporated additional references and explanations to support the clinical relevance of using different ABI methods.

“These approaches aim to balance diagnostic sensitivity and specificity while enhancing the prediction of cardiovascular risk and lower-limb function. Although the highest ankle pressure remains the standard for population studies due to its specificity and comparability, the lowest pressure improves sensitivity for detecting milder or asymptomatic cases. The average pressure has shown stronger associations with functional performance, such as walking speed and leg strength. Each method yields different PAD prevalence estimates and identifies distinct risk profiles.”

7. Page 10, Line 68: The prevalence of PAD in Peruvian hospitals is cited as 18.9% (reference 9), but the cited study focuses on pulsioximetry, not ABI..

Response: We have corrected the citation to clarify that the data were based on pulsioximetry and not ABI.

8. Page 12, Lines 101-103: The objectives mention determining PAD and AC frequency using ABI but do not specify the three methods (highest, lowest, average), misaligning with the title and methods section (e.g., Page 13, Lines 121-125).

We thank the reviewer for this valuable observation. In response, we have revised the objectives in both the Abstract and the Introduction to explicitly state that the aim of the study was to determine the frequency of peripheral arterial disease (PAD) and arterial calcification (AC) using three different methods of ABI calculation: the highest, lowest, and average systolic ankle pressures. This modification ensures alignment with the study title, methodology, and analytical approach.

9. Page 13, Lines 105-110: The study is described as cross-sectional, but it does not clarify that it is a secondary analysis of existing data from the Foot at Risk Program.

We thank the reviewer for this helpful observation. We agree with the need to clarify the data source and have updated the Study Design subsection of the Methods section accordingly. The current version now explicitly states that this is a secondary analysis of de-identified clinical data collected prospectively through the Foot at Risk Program at Hospital María Auxiliadora.

10. Page 13, Lines 111-118: Inclusion criteria mention records with complete ABI data, but exclusion criteria (e.g., lesions, severe pain) are listed without explaining their impact on ABI feasibility or prevalence estimates.

We thank the reviewer for this important observation. We have clarified in the revised Methods section that exclusion criteria such as active foot lesions, severe lower-limb pain, poorly controlled blood pressure, or marked edema were applied because these conditions compromise the feasibility and accuracy of ABI measurement in routine clinical practice. However, we also acknowledge that these exclusions may introduce selection bias, as they could systematically exclude patients with more advanced vascular or metabolic disease. This, in turn, may lead to an underestimation of the true prevalence of peripheral arterial disease (PAD) or arterial calcification (AC). We have now explicitly discussed this potential limitation in both the Population and Sample subsection and the Limitations section of the manuscript.

11. Page 17, Lines 208-211: Of 1,019 patients, 369 were excluded for incomplete ABI data, but no reasons (e.g., technical issues, patient factors) are provided.

We thank the reviewer for this comment. We have now detailed the reasons for incomplete ABI data in the revised manuscript. Of the 1,019 patients initially assessed, 376 were excluded: 5 due to a history of major amputation and 2 due to a history of stroke. The remaining 369 were excluded because of incomplete ABI data. Among these, 271 patients had no ABI measurement, mostly due to edema, hypertensive urgency, or acute foot ulcer (n=246), with 25 cases undocumented. Five patients had only one ABI, with reasons documented in 4 cases (clinical conditions as above) and unknown in 1 case. Eighty-one patients had two ABIs, with 70 due to clinical conditions and 11 undocumented. Twelve patients had three ABIs, with 10 due to clinical conditions and 2 undocumented. These details have been added to the Methods section for clarity.

12. Page 13, Lines 120-125: ABI methods are defined, but the measurement of systolic pressures (e.g., number of readings, operator consistency) is not detailed.

We appreciate the reviewer’s insightful comment and have revised the Methods section to provide greater detail. As part of standard clinical practice within the At-Risk Foot Program, only one systolic pressure reading per artery was obtained during each evaluation. Although repeated measurements were not performed and no formal assessment of intra- or inter-observer variability was conducted, all measurements were carried out by two endocrinologists with over 10 years of clinical experience in vascular evaluation, which contributes to consistency and reliability of the measurements. We also acknowledge this limitation and have explicitly added the absence of repeated measurements as a potential source of measurement bias in the study’s Limitations section.

13. Page 14, Lines 139-144: Poor metabolic control (HbA1c ≥ 7%) is defined, but its role in the analysis (e.g., as a confounder or outcome modifier) is unclear.

We thank for this observation. However, given the descriptive and cross-sectional nature of the study, poor metabolic control (defined as HbA1c ≥ 7%) was not modeled as a confounder or effect modifier, since the objective was not to assess causal relationships. Rather, glycemic control was included as a relevant clinical characteristic to describe the population and explore its association with the distribution of ABI categories. We acknowledge that causal inference would require longitudinal data and temporality, which are beyond the scope of this analysis.

14. Page 14, Lines 152-155: ABI measurement tools (sphygmomanometer, Doppler) are listed, but the protocol (e.g., cuff size, measurement sequence) and personnel training standardization are not described.

We thank for this observation. In response, we have expanded the manuscript to include a brief protocol describing how ABI measurements were performed. All assessments followed a standardized clinical procedure: patients rested in the supine position for at least 10 minutes prior to measurement; appropriate cuff sizes were selected based on limb circumference; and systolic pressures were recorded sequentially at both brachial arteries and at the dorsalis pedis and posterior tibial arteries of both ankles. The highest brachial pressure was used as the denominator, and each ankle artery was measured once, in accordance with routine clinical practice. Although no formal intra- or inter-observer agreement assessment was performed, all measurements were conducted by two endocrinologists with over 10 years of experience in vascular and diabetic foot evaluation, which ensures procedural consistency. These additions have been incorporated into the revised Methods section.

15. Page 15, Lines 164-165: No imputation for missing data is noted, but the extent and pattern of missingness (e.g., 369 incomplete ABI records) are not analyzed.

We thank the reviewer for this important observation. As noted, no imputation was performed for missing data, and records with incomplete ABI measurements (n = 369) were excluded from the analysis. While the reasons for missing ABI data were not systematically recorded—likely including technical limitations, patient discomfort, or incomplete documentation—we acknowledge that not characterizing these excluded cases may introduce selection bias. We have now added this point explicitly to the Limitations section, noting that the absence of an analysis of excluded individuals limits our understanding of the extent and potential pattern of missingness and may affect the generalizability of the findings

16. Page 13-16: Potential biases (e.g., selection bias from excluding incomplete records, information bias from self-reported variables like diabetes duration) are not identified or addressed.

We thank the reviewer for this insightful comment. In response, we have revised the Limitations section to explicitly address potential sources of bias. First, we acknowledge the possibility of **selection bias** due to the exclusion of patients with incomplete ABI data, which may have led to an underrepresentation of individuals with more advanced disease or technical challenges in ABI measurement. Second, we recognize the risk of **information bias**, particularly related to self-reported variables such as diabetes duration and history of complications, which may be affected by recall inaccuracies or inconsistent documentation. While these limitations are inherent to secondary analyses of routine clinical data, we believe that their inclusion in the revised manuscript enhances transparency and helps contextualize the findings.

17. Page 16, Lines 189-192: Adjustments in Poisson regression are made for variables with p < 0.2, but the rationale for this threshold and the specific confounders adjusted for are not specified.

We thank the reviewer for this observation. We have clarified in the Methods section that variables with p < 0.20 in the bivariate analysis were considered candidates for the multivariable Poisson regression model. This threshold is frequently used in epidemiological and prognostic modeling to avoid excluding potential confounders that may have weaker univariate associations but remain important in multivariable adjustment. This approach is supported by Steyerberg and Van Calster (2020), who recommend using higher p-value thresholds in diagnostic and prognostic modeling to improve model stability and reproducibility. The final model was adjusted for: age group, sex, education level, diabetes duration, hypertension history, coronary artery disease history, peripheral neuropathy, diabetic retinopathy, BMI category, systolic blood pressure, LDL cholesterol, triglycerides, and HbA1c. This information has been added to the Statistical Analysis subsection for clarity.

Reference:

Steyerberg EW, Van Calster B. Redefining significance and reproducibility for medical research: A plea for higher P-value thresholds for diagnostic and prognostic models. Eur J Clin Invest. 2020;50:e13229. doi:10.1111/eci.13229

18. Page 16, Lines 185-192: Poisson regression with robust variance is used, but the choice over other methods (e.g., logistic regression) and its application (e.g., model assumptions) are not explained.

We thank the reviewer for this thoughtful observation. In response, we have revised the Methods section to clarify the rationale for selecting Poisson regression with robust variance. This method was chosen over logistic regression because our outcome variables (PAD and AC) had relatively high prevalence. In such cases, logistic regression can lead to an overestimation of effect sizes when odds ratios are interpreted as approximations of prevalence ratios.

Although log-binomial regression is another method capable of directly estimating prevalence ratios, we initially attempted to use it; however, the model failed to converge, which is a known limitation of log-binomial models when the data structure is complex or prevalence is high. For this reason, we opted for modified Poisson regression with robust variance estimation, which produces consistent and interpretable estimates of prevalence ratios (PRs) and is less susceptible to convergence problems (Barros & Hirakata, 2003; Zou, 2004).

We also added details in the revised manuscript indicating that the model assumes independent observations, binary outcomes, and uses robust standard errors to account for overdispersion and violation of Poisson distribution assumptions. This modeling approach is widely accepted in clinical epidemiology, especially for cross-sectional analyses with non-rare outcomes.

These clarifications have been included in the “Analysis Plan” subsection and the limitations of the approach are now discussed in the manuscript.

References:

Barros, A. J. D., & Hirakata, V. N. (2003). Alternatives for logistic regression in cross-sectional studies: an empirical comparison of models that directly estimate the prevalence ratio. BMC Medical Research Methodology, 3, 21. https://doi.org/10.1186/1471-2288-3-21

Zou, G. (2004). A modified Poisson regression approach to prospective studies with binary data. American Journal of Epidemiology, 159(7), 702–706. https://doi.org/10.1093/aje/kwh090

19. Page 15, Lines 169-174: The ABI calculation algorithm prioritizes PAD over AC, but this hierarchical approach’s impact on AC prevalence (e.g., underreporting in mixed cases) is not acknowledged.

Response:

We thank the reviewer for this important observation. In response, we have clarified in the Methods section that the classification algorithm was hierarchical: in cases where one limb had an ABI < 0.9 (PAD) and the contralateral limb had an ABI > 1.3 (AC), the patient was classified under the PAD category. This decision was based on prior literature emphasizing the clinical relevance of PAD as a priority condition for detection and management.

However, we agree that this approach may have underestimated the true prevalence of arterial calcification (AC), particularly in individuals with asymmetrical vascular involvement. As suggested, this limitation has now been acknowledged explicitly in the Discussion section, under the study limitations. We also added a clarification that future studies using limb-level analyses could provide more precise estimates of overlapping PAD and AC phenotypes.

Results

20. Page 17, Lines 208-211: The participant flow excludes 369 subjects with incomplete ABI data, but no comparison of excluded vs. included subjects is provided.

Thank you for your comment. In total, 376 participants were excluded from the final analysis: 369 due to incomplete ABI measurements, 5 due to major lower-limb amputation, and 2 due to a history of stroke. To evaluate the potential for selection bias, we compared included (n = 643) and excluded participants (n = 376) across key demographic, clinical, and laboratory variables. The two groups were comparable in terms of sex, diabetes duration, BMI, LDL-c levels, and medication regimen. However, included participants were slightly older (mean 61.4 vs. 59.3 years, p = 0.002), had lower fasting glucose levels (p = 0.024), lower prevalence of hypertension (p < 0.001), and higher prevalence of neuropathy (p = 0.001). These differences are now reported in Supplementary Table S1, and the potential impact of selection bias on the study’s findings has been discussed in the revised Discussion section.

21. Page 18, Lines 219-228: Baseline characteristics (e.g., 69.8% female, mean age 61.4) are reported, but missing data for variables like HbA1c (325/643 records) are not quantified or analyzed for bias.

We appreciate the reviewer’s valuable observation. In response, we clarify that the original table of baseline characteristics already included a column reporting the number of available observations (N available) per variable. To enhance transparency regarding data completeness, we have now incorporated the proportion of available data (% of total, N = 643) for each variable. This addition allows readers to more readily assess the extent of missingness across variables. While a formal sensitivity or bias analysis was not conducted for incomplete data, we explicitly acknowledge this limitation in the Discussion section.

22. Page 20, Lines 240-242: Prevalence estimates differ by ABI method (e.g., PAD: 7.8% vs. 28.2%), but the statistical significance of these differences is not tested.

We thank the reviewer for this insightful observation. In response, we have now tested the statistical significance of the differences in prevalence estimates of peripheral arterial disease (PAD) and arterial calcification (AC) obtained using the three ABI calculation methods (highest, average, and lowest ankle systolic pressure). Given that each method classifies the same set of patients into three mutually exclusive categories (normal, PAD, or AC), we applied Bowker’s test of symmetry to assess whether these classification differences were statistically significant. This test is an appropriate extension of McNemar’s test for paired categorical data with more than two categories and evaluates whether there is symmetry in the classification distribution between paired methods.

The results of the symmetry test demonstrated significant asymmetry between the methods, indicating that the differences in PAD and AC prevalence across ABI calculation strategies are not due to random variation. These findings have been incorporated into the revised analysis plan and will be discussed in the Results and Discussion sections to support the methodological implications of choosing a specific ABI calculation approach.

23. Page 21, Lines 257-269: Adjusted prevalence ratios (aPRs) for PAD (e.g., aPR 8.2 for age > 75) are reported, but confidence intervals are wide (e.g., 1.11-60.1).

We appreciate the reviewer’s observation. We acknowledge that some adjusted prevalence ratios (aPRs), particularly those related to older age groups, show wide confidence intervals. This is likely due to the limited number of PAD cases in certain subgroups, which reduces the precision of the estimates. Additionally, although the total sample size is adequate, the stratification by age, ABI method, and PAD category resulted in smaller cell counts for specific categories.

We have now emphasized this limitation in the Discussion section, noting that the wide confidence intervals reflect statistical imprecision and should be interpreted cautiously. However, the direction and magnitude of the association remain clinically plausible and consistent with prior literature indicating that older age is a strong risk factor for peripheral arterial disease.

We have also clarified in the Results section that the estimates should not be overinterpreted, especially when the confidence intervals suggest considerable uncertainty.

24. Page 25, Lines 280-294: AC associations (e.g., aPR 3.79 for diabetes > 20 years) are presented, but the lack of adjustment for key confounders like age in all models is not justified.

We thank the reviewer for this valuable observation. We would like to clarify that age was in fact included as an adjustment variable in all multivariate models, as shown in the results table. Specifically, age categories (<60, 60–74.9, ≥75 years) were entered into the regression models regardless of their univariate significance, based on their known clinical relevance as a confounding factor in peripheral arterial disease and vascular calcification.

In the final adjusted model for arterial calcification (AC), age was retained and reported for each ABI method. For example, under the lowest ABI criterion, the age group 60–74.9 years showed an adjusted prevalence ratio (aPR) of 0.57 (95% CI: 0.37–0.87, p = 0.010), and for those ≥75 years the aPR was 1.07 (95% CI: 0.59–1.92, p = 0.810). These results are clearly presented in the corresponding table of multivariable analyses.

Discussion

25. Page 29, Lines 304-319: The summary of PAD prevalence aligns with objectives, but the interpretation (e.g., “optimize risk identification”) overstates findings without evidence of diagnostic accuracy.

We appreciate this insightful observation. In response, we have revised the interpretation of the results to avoid overstatement and to remain aligned with the descriptive and comparative nature of our cross-sectional study. Specifically, we have removed the phrase "optimize risk identification" from the discussion and conclusion sections, as our design does not evaluate diagnostic accuracy or predictive performance.

26. Page 31, Lines 372-381: The claim that lowest ABI identifies more at-risk individuals assumes a link to mortality outcomes not tested in this study.

We appreciate the reviewer’s comment and agree that our study did not evaluate mortality or other hard outcomes directly. The statement regarding the use of the lowest SAP criterion for risk stratification and the highest SAP criterion for severity assessment and post-revascularization follow-up was not intended to imply that these associations were tested or confirmed in our analysis. Rather, this sentence was meant to summarize the recommendations from the cited European Society for Vascular Surgery (ESVS) 2024 Clinical Practice Guidelines (Nordanstig et al., 2024), which provide these criteria based on prior evidence. To avoid any misinterpretation, we have revised the text to explicitly state that this recommendation derives from the guideline and is not a finding of our study.

27. Page 32, Lines 389-400: Selection bias from hospital-based sampling and incomplete records is mentioned, but its direction and magnitude (e.g., overestimating PAD prevalence) are not explored.

Thank you for this comment. We agree that potential selection bias arising from hospital-based sampling and the exclusion of incomplete records could affect the estimated prevalence of PAD. This bias may lead to an overestimation of PAD prevalence, as individuals attending tertiary-level hospital programs—such as the Foot at Risk Clinic—may present with more advanced or symptomatic disease compared to the general population with diabetes. We have clarified this in the Discussion section, acknowledging both the direction (possible overestimation) and the limited ability to quantify its magnitude due to the absence of comparative data from excluded or community-based population

28. Page 32, Lines 389-391: Generalizability is questioned due to the hospital setting, but no discussion addresses how the Peruvian context (e.g., healthcare access) affects external validity.

Thank you for your valuable comment. We agree that the generalizability of our findings is limited by the hospital-based nature of the study, conducted within a tertiary care Foot at Risk Program. We have now expanded the Discussion section to explicitly address this issue. Studies based on patients attending hospital settings, particularly those referred for specialized diabetic foot care, often lack external validity due to selection bias toward individuals with more advanced disease or greater healthcare access. While these settings provide valuable insights into high-risk populations, they may not fully represent the general diabetic population in the community.

We recognize the importance of conducting population-based studies to establish prevalence estimates with broader applicability. However, such studies require greater resources, including complex sampling strategies, population-level recruitment, and wider availability of Doppler devices and trained personnel to conduct ABI measurements. These limitations are particularly relevant in low- and middle-income countries like Peru, where barriers to access and disparities in healthcare infrastructure can impact the feasibility of such designs. We have added this clarification to the manuscript.

29. Page 29, Lines 310-319: Comparisons to US and French studies (references 27, 28) are made, but differences in population (e.g., multiethnic vs. Peruvian) and methodology are not critically analyzed.

Thank you for your comment. We agree that differences in population characteristics and methodology should be more explicitly addressed when comparing our results to those of U.S. and French studies (references 27 and 28). We have now expanded the Discussion section to reflect this. Specifically, the U.S. study by Allison et al. (reference 27) was conducted in a multiethnic general population with complex sampling, and the French cohort study (reference 28) was embedded in a primary care context with broader access to vascular screening. In contrast, our study population consisted exclusively of Peruvian adults with type 2 diabetes attending a tertiary hospital-based foot care program, which may represent a group with higher risk and disease burden. Furthermore, the methodologies differ in terms of ABI measurement protocols, classification thresholds, and definitions of peripheral arterial disease and arterial calcification. These methodological variations and population differences could explain the higher prevalence of PAD found in our study and highlight the need for context-specific screening and prevention strategies.This comparative reflection has now been integrated into the revised Discussion section

30. Page 33, Lines 405-411: Future research is suggested, but the discussion lacks specific gaps (e.g., validation against outcomes) or hypotheses, reducing its utility.

Thank you for this valuable comment. We agree that our original discussion lacked specificity regarding research gaps and future directions. We have now revised the Discussion section to explicitly outline the need for studies that validate the prognostic utility of different ABI calculation methods by linking them with hard clinical outcomes such as cardiovascular events, limb ischemia, and mortality. Additionally, we suggest that future research should evaluate the diagnostic accuracy, reproducibility, and cost-effectiveness of each method, particularly in low-resource settings. This will allow the development of context-appropriate guidelines for peripheral arterial disease screening among patients with diabetes.

Additional Comments

31. Throughout Manuscript: Terminology for ABI methods varies (e.g., “highest pressure criterion” on Page 20 vs. “high ABI” on Page 21), risking confusion.

We appreciate the reviewer’s observation. To avoid any ambiguity and ensure consistency throughout the manuscript, we have standardized the terminology for ABI calculation methods. We now uniformly refer to:

- Highest systolic ankle pressure criterion (highest value between dorsalis pedis and posterior tibial arteries)
- Average systolic ankle pressure criterion (mean of both arteries)
- Lowest systolic ankle pressure criterion (lowest value between both arteries)

This terminology is used consistently in all sections, tables, and figures, replacing less precise terms such as “high ABI” to prevent misinterpretation.

32. Page 33, Line 407: “CA” is used instead of “AC” (consistent with Abstract, Line 45), indicating a persistent typographical error.

Thank you for pointing out this inconsistency. We have carefully reviewed the entire manuscript and standardized the abbreviation to “AC” to ensure consistency and clarity in referring to arterial calcification.

33. Page 22-28, Tables 3-4: Adjusted models include variables with p < 0.2 from crude analysis, but multicollinearity or model overfitting (e.g., many covariates for 643 subjects) is not addressed.

We appreciate the reviewer’s observation regarding potential multicollinearity in the adjusted models. In response, we evaluated collinearity in each of the six adjusted Poisson regression models (i.e., three models for peripheral arterial disease [PAD] and three for arterial calcification [AC]) using the **variance inflation factor (VIF)** with the **uncentered option**, as recommended in epidemiological modeling practices. None of the independent variables included in the final models presented a VIF greater than 10, indicating no concerning level of multicollinearity. This methodological detail has been added to the Statistical Analysis section of the manuscript.

## Reviewer #2 Comments and Responses

Major Comments:

34. Study Design and Methodology:

    The manuscript states that the study is observational, cross-sectional, and descriptive. However, it would benefit from a clearer justification for choosing this design over a longitudinal approach, which could provide insights into the progression of PAD and AC over time.

We thank the reviewer for this thoughtful observation. We agree that a longitudinal design could offer valuable insights into the progression and temporal dynamics of peripheral arterial disease (PAD) and arterial calcification (AC). However, the current study was conceptualized as a **cross-sectional secondary analysis** of routinely collected clinical data from the Foot at Risk Program at a public hospital in Peru. This design was selected based on the availability of data and the exploratory objective of comparing the **prevalence estimates of PAD and AC** across three different ankle-brachial index (ABI) calculation methods.

Given the limited resources and infrastructure typically available in low- and middle-income healthcare settings, implementing a longitudinal study would require substantial additional funding, patient follow-up mechanisms, and repeated ABI measurements—conditions that were not feasible within the scope of this project. Nonetheless, we agree that our findings underscore the need for future **prospective studies** to evaluate the predictive validity of each ABI method in relation to vascular outcomes, including mortality and limb complications.

We have added a statement in the Discussion section to clarify this rationale.

35. The exclusion criteria (e.g., major amputation, stroke, incomplete ABI data) are appropriate, but the high exclusion rate (369/1019, ~36%) due to incomplete ABIs raises concerns about potential selection bias. Please discuss how this might affect the generalizability of the findings.

To address concerns regarding potential selection bias from the exclusion of 376 participants (~36%) due to incomplete ABI measurements, we compared baseline characteristics between included and excluded individuals. No significant differences were observed in sex distribution (69.8% vs. 68.6% female, p = 0.685), diabetes duration (mean 8.9 vs. 8.7 years, p = 0.70), or LDL-cholesterol levels (mean 120.5 mg/dL in both groups, p = 0.98). However, included participants were slightly older (mean age 61.4 vs. 59.3 years, p = 0.002), had a higher prevalence of hypertension (33.2% vs. 19.0%, p < 0.001), more frequent peripheral neuropathy (81.3% vs. 71.8%, p = 0.001), and lower glycemia levels (mean glucose 172.9 vs. 188.9 mg/dL, p = 0.024). There were also differences in diabetes treatment patterns, with a greater proportion of insulin use in the included group (32.2% vs. 25.0%, p = 0.027). These differences suggest that included participants may represent a slightly more complex metabolic and cardiovascular risk profile, potentially leading to a modest overestimation of PAD and AC prevalence in our findings. These limitations have been acknowledged in the revised manuscript.

36. ABI Calculation Methods:

    The three ABI calculation methods (highest, lowest, and average systolic pressure) are clearly described, but the rationale for selecting these specific methods could be expanded. Are there clinical guidelines or prior studies that support these choices?

Thanks for flagging this—we’ve expanded the rationale in the **Introduction** to make our choices explicit. Briefly: we use the **highest ankle systolic pressure** because this is the guideline-standard ABI for screening and comparability, minimizing false positives from segmental disease (ESVS 2024). We also report the **lowest ankle pressure** to increase sensitivity and capture additional at-risk patients—an approach supported for risk stratification in high-risk groups such as diabetes (ESVS 2024; Filho et al., 2022). Finally, we include the **average pressure** because prior work suggests its prognostic performance is comparable when method-appropriate thresholds are applied, adding nuance for functional and risk prediction analyses (Le Bivic et al., 2019).

**References**

- Nordanstig J, Behrendt CA, Baumgartner I, et al. ESVS 2024 Clinical Practice Guidelines on the Management of Asymptomatic Lower Limb Peripheral Arterial Disease and Intermittent Claudication. Eur J Vasc Endovasc Surg. 2024;67:9–96. doi:10.1016/j.ejvs.2023.08.067
- Filho A, Sartipy F, Lundin F, et al. Impact of Ankle-Brachial Index Calculations for Peripheral Arterial Disease Prevalence and as a Predictor for Cardiovascular Risk. Eur J Vasc Endovasc Surg. 2022;64(3):370–377. doi:[10.1016/j.ejvs.2022.05.001](https://doi.org/10.1016/j.ejvs.2022.05.001)
- Le Bivic L, Magne J, Guy-Moyat B, et al. The intrinsic prognostic value of the ankle–brachial index is independent from its mode of calculation. Vasc Med. 2019;24(1):23–31. doi:[10.1177/1358863X18807003](https://doi.org/10.1177/1358863X18807003)

37. The manuscript mentions that the lowest ABI criterion increases sensitivity but decreases specificity. This trade-off should be discussed in greater depth, particularly in terms of clinical decision-making (e.g., how might false positives/negatives impact patient outcomes?).

We appreciate the reviewer’s observation regarding the need to elaborate on the trade-off between increased sensitivity and decreased specificity when using the lowest ABI criterion. We have now expanded the discussion to address the clinical implications of this trade-off, supported by recent literature and the 2024 European Society for Vascular Surgery (ESVS) Clinical Practice Guidelines.

Using the lowest ABI criterion increases sensitivity—allowing more true cases of peripheral arterial disease (PAD) to be detected—but decreases specificity, leading to more false positives. As highlighted in the ESVS 2024 guidelines, this approach may be clinically justified in high-risk populations, such as individuals with diabetes, where the consequences of missed diagnoses outweigh the harms of overdiagnosis (Nordanstig et al., 2024). However, false positives can result in unnecessary further testing, increased healthcare costs, and patient anxiety, while false negatives may delay diagnosis and treatment, increasing the risk of progression to critical limb ischemia (Da Fonseca Cerqueira et al., 2024).

We now explicitly state that the choice of ABI calculation method should be guided by the clinical context and patient risk profile. In specialized or high-prevalence cohorts, such as our hospital-based population with type 2 diabetes, prioritizing sensitivity by using the lowest ABI may be appropriate for early risk identification, provided it is combined with confirmatory testing and integrated into a broader diagnostic framework, as recommended by the ESVS 2024 guidelines.

References

1. Nordanstig J, Behrendt CA, Baumgartner I, Belch J, Bäck M, Fitridge R, et al. Editor’s Choice – European Society for Vascular Surgery (ESVS) 2024 Clinical Practice Guidelines on the Management of Asymptomatic Lower Limb Peripheral Arterial Disease and Intermittent Claudication. *Eur J Vasc Endovasc Surg*. 2024;67: 9–96. doi:10.1016/j.ejvs.2023.08.067
2. Da Fonseca Cerqueira M, Bastos N, Da Silva D, Gregori D, Magalhães L, Pimentel M. Accuracy of ankle-brachial index in screening for peripheral arterial disease in people with diabetes. *PLOS ONE*. 2024;19(8):e0309083. doi:10.1371/journal.pone.0309083
38. Prevalence Estimates:

The reported prevalence of PAD varies widely (7.8% to 28.2%) depending on the ABI method. While this variability is a key finding, the discussion should address why such discrepancies exist and which method might be most appropriate for specific clinical scenarios (e.g., screening vs. diagnostic confirmation).

We appreciate this comment and have expanded the **Discussion** to address the observed variability in PAD prevalence (7.8% to 28.2%) across ABI calculation methods. This discrepancy arises from inherent methodological differences: using the **highest ankle systolic pressure**—as recommended by current guidelines—reduces false positives by minimizing the impact of segmental disease, but may miss cases with isolated arterial lesions (lower sensitivity) (Nordanstig et al., 2024). Conversely, the **lowest ankle pressure** increases sensitivity, capturing additional at-risk individuals, but decreases specificity, potentially leading to overdiagnosis (Filho et al., 2022). The **average pressure** approach shows intermediate prevalence estimates and has been explored for its prognostic comparability when thresholds are method-specific (Le Bivic et al., 2019).

From a clinical perspective, we note that the highest ankle pressure method is most suitable for **screening in general populations** where specificity and comparability with existing literature are critical. The lowest ankle pressure may be more appropriate in **high-risk populations** (e.g., diabetes, prior cardiovascular disease) where the priority is to avoid missed diagnoses, even at the cost of more false positives. The average method, while less common in guidelines, could be valuable in **research settings** aimed at prognostic modeling or functional assessments.

**References**

- Nordanstig J, Behrendt CA, Baumgartner I, et al. ESVS 2024 Clinical Practice Guidelines on the Management of Asymptomatic Lower Limb Peripheral Arterial Disease and Intermittent Claudication. Eur J Vasc Endovasc Surg. 2024;67:9–96. doi:10.1016/j.ejvs.2023.08.067
- Filho A, Sartipy F, Lundin F, et al. Impact of Ankle-Brachial Index Calculations for Peripheral Arterial Disease Prevalence and as a Predictor for Cardiovascular Risk. Eur J Vasc Endovasc Surg. 2022;64(3):370–377. doi:[10.1016/j.ejvs.2022.05.001](https://doi.org/10.1016/j.ejvs.2022.05.001)
- Le Bivic L, Magne J, Guy-Moyat B, et al. The intrinsic prognostic value of the ankle–brachial index is independent from its mode of calculation. Vasc Med. 2019;24(1):23–31. doi:[10.1177/1358863X18807003](https://doi.org/10.1177/1358863X18807003)

39. The prevalence of AC (11% to 18.2%) is less variable but still noteworthy. The authors should clarify whether AC was assessed independently of PAD or if overlapping cases were excluded.

We appreciate the comment. In our study, arterial calcification (AC) was assessed using a hierarchical model: peripheral arterial disease (PAD) was diagnosed first using an ABI cutoff of < 1.3, and AC (ABI > 1.3) was evaluated only in cases without PAD. However, we acknowledge that a minimal overlap between both conditions may occur. In our sample, the proportion of subjects with a simultaneous diagnosis of PAD and AC was low (ranging from 2.0% to 7.7% of PAD cases, depending on the ABI calculation method), suggesting that the vast majority of AC diagnoses were made independently of PAD.

40. Associations with Clinical Variables:

    The association of PAD with older age and AC with longer diabetes duration is consistent with prior literature. However, the manuscript could better contextualize these findings by comparing them to similar studies in other populations.

We agree that situating our results within the international literature strengthens their interpretation. Our findings—that PAD is associated with older age and AC with longer diabetes duration—are consistent with reports from Asia, Europe, and Latin America (Rhee et al., 2007; Escobar et al., 2011; Arora et al., 2024; Harjutsalo et al., 2021; Ascencio et al., 2024). Globally, older age remains a robust determinant of PAD, while longer diabetes duration is a key predictor of AC, supporting targeted vascular screening in these high-risk subgroups.

**References**

1. Rhee, S., Guan, H., Liu, Z., Cheng, S., Waspadji, S., Palmes, P., Tai, T., Suwanwalaikorn, S., & Kim, Y. (2007). Multi-country study on the prevalence and clinical features of peripheral arterial disease in Asian type 2 diabetes patients at high risk of atherosclerosis. Diabetes Research and Clinical Practice, 76(1), 82–92. <https://doi.org/10.1016/j.diabres.2006.07.029>
2. Arora, E., Regan, R., Surendra, V., & Arumugam, A. (2024). Prevalence of peripheral arterial disease among individuals with type 2 diabetes mellitus in India – A systematic review and meta-analysis. Diabetes & Metabolic Syndrome, 18(9), 103124. <https://doi.org/10.1016/j.dsx.2024.103124>
3. Escobar, C., Blanes, I., Ruiz, A., Vinuesa, D., Montero, M., Rodríguez, M., Barberá, G., & Manzano, L. (2011). Prevalence and clinical profile and management of peripheral arterial disease in elderly patients with diabetes. European Journal of Internal Medicine, 22(3), 275–281. <https://doi.org/10.1016/j.ejim.2011.02.001>
4. Harjutsalo, V., Barlovič, D., Gordin, D., Forsblom, C., King, G., & Groop, P. (2021). Presence and determinants of cardiovascular disease and mortality in individuals with type 1 diabetes of long duration: The FinnDiane 50 Years of Diabetes Study. Diabetes Care, 44(9), 1885–1893. <https://doi.org/10.2337/dc20-2816>
5. Ascencio, A., Carmona, E., Farías, J., Medina, D., Salas, R., & Reyna, L. (2024). Prevalence of peripheral arterial disease and principal associated risk factors in patients with type 2 diabetes mellitus: The IDON-Peripheral Arterial Disease Study. Diabetology, 5(2), 15. <https://doi.org/10.3390/diabetology5020015>
41. The inverse association between obesity and PAD (using the high ABI method) is intriguing but counterintuitive. The authors should explore potential explanations (e.g., confounding by other metabolic factors) or limitations (e.g., small sample size in subgroups).

We appreciate the reviewer’s comment and acknowledge that the inverse association between obesity and PAD observed with the high ABI criterion is counterintuitive and consistent with the “obesity paradox” described in PAD (Lempesis et al., 2023). Possible explanations include earlier diagnosis and more intensive risk factor control in obese patients (Zhong et al., 2024), BMI’s limited ability to capture body fat distribution (Cronin et al., 2013), and reverse causality, where lower BMI reflects disease-related weight loss or frailty (Lin et al., 2022). Biological mechanisms, such as inflammatory profile differences, may also contribute (Huang et al., 2024). Given our use of BMI as the sole obesity metric and the limited subgroup sample size, these findings should be interpreted with caution, and future studies should include body composition measures.

**References**

1. Lempesis, I., Varrias, D., Sagris, M., Attaran, R., Altin, E., Bakoyiannis, C., Palaiodimos, L., Dalamaga, M., & Kokkinidis, D. (2023). Obesity and Peripheral Artery Disease: Current Evidence and Controversies. Current Obesity Reports, 1–16. <https://doi.org/10.1007/s13679-023-00510-7>
2. Zhong, Q., Yunus, R., Sohail, M., Saeed, S., Rehman, T., Khan, A., Russ, E., Schermerhorn, M., Mahmood, F., & Matyal, R. (2024). Association of Body Surface Area Versus Body Mass Index on Outcomes in Peripheral Arterial Disease. Annals of Vascular Surgery. <https://doi.org/10.1016/j.avsg.2024.07.099>
3. Lin, D., Lo, H., Yu, A., Lee, J., & Chien, K. (2022). Mortality risk in patients with underweight or obesity with peripheral artery disease: a meta-analysis including 5,735,578 individuals. International Journal of Obesity, 46, 1425–1434. <https://doi.org/10.1038/s41366-022-01143-x>
4. Huang, X., Pang, S., Zhang, T., & Huang, C. (2024). Assessment of causal associations between obesity and peripheral artery disease: a bidirectional Mendelian randomization study. Frontiers in Cardiovascular Medicine, 11. <https://doi.org/10.3389/fcvm.2024.1332530>
5. Cronin, O., Morris, D., Walker, P., & Golledge, J. (2013). The association of obesity with cardiovascular events in patients with peripheral artery disease. Atherosclerosis, 228(2), 316–323. <https://doi.org/10.1016/j.atherosclerosis.2013.03.002>
42. Limitations:

    The hospital-based design limits generalizability, as acknowledged by the authors. However, the discussion should also address how the study population (e.g., predominantly female, mean age 61.4 years) might influence the results.

We agree that the demographic characteristics of our predominantly female, hospital-based cohort (mean age 61.4 years) may have influenced the observed prevalence estimates and associations. PAD prevalence increases with age, and sex-related differences in ABI values and disease presentation have been described, with women often showing lower ABI values and a higher prevalence of borderline PAD, potentially due to smaller arterial diameters and hormonal influences (Harjutsalo et al., 2021; Aboyans et al., 2018; Song et al., 2019). In our outpatient setting, women are more frequently represented in preventive evaluations such as foot-at-risk screening, whereas the proportion of men tends to rise once complications develop. This pattern is consistent with evidence that women with type 2 diabetes generally demonstrate greater engagement in preventive care and higher adherence to medical appointments compared with men (Mesa et al., 2018). Although PAD has historically been considered more prevalent in men, contemporary data indicate comparable or even higher prevalence in nonwhite women, who may present with atypical or asymptomatic disease and experience worse cardiovascular outcomes (Srivaratharajah & Abramson, 2018). These demographic and care-seeking patterns likely contributed to the female predominance in our sample and should be considered when interpreting the generalizability of our findings.

**References**

1. Harjutsalo, V., Barlovič, D., Gordin, D., Forsblom, C., King, G., & Groop, P. (2021). Presence and Determinants of Cardiovascular Disease and Mortality in Individuals With Type 1 Diabetes of Long Duration: The FinnDiane 50 Years of Diabetes Study. Diabetes Care, 44, 1885–1893. <https://doi.org/10.2337/dc20-2816>
2. Arora, E., Regan, R., Surendra, V., & Arumugam, A. (2024). Prevalence of peripheral arterial disease among individuals with type 2 diabetes mellitus in India - A systematic review and meta-analysis. Diabetes & Metabolic Syndrome, 18(9), 103124. <https://doi.org/10.1016/j.dsx.2024.103124>
3. Song, P., Rudan, D., Zhu, Y., Fowkes, F., Rahimi, K., Fowkes, F., & Rudan, I. (2019). Global, regional, and national prevalence and risk factors for peripheral artery disease in 2015: an updated systematic review and analysis. The Lancet Global Health, 7(8), e1020–e1030. <https://doi.org/10.1016/S2214-109X(19)30255-4>
4. Mesa MS. Health Care Disparities Between Men and Women With Type 2 Diabetes. Prev Chronic Dis. 2018;15:E46. https://doi:10.5888/pcd15.170120.
5. Srivaratharajah K, Abramson BL. Women and Peripheral Arterial Disease: A Review of Sex Differences in Epidemiology, Clinical Manifestations, and Outcomes. Can J Cardiol. 2018;34(4):356–361. https://doi:10.1016/j.cjca.2018.01.009.
43. The underreporting of AC due to prioritization of PAD in the diagnostic algorithm is a significant limitation. The authors should discuss how this might have affected the observed prevalence and associations.

We agree that prioritizing PAD over AC in our hierarchical diagnostic algorithm likely led to underreporting of AC. Participants meeting PAD criteria (<0.9) were classified as PAD even if they also met AC criteria (>1.3), resulting in 2.0–7.7% “overlap cases” being coded exclusively as PAD. This may have slightly underestimated AC prevalence—particularly in older adults and those with long-standing diabetes—attenuated AC–risk factor associations, and modestly overestimated PAD prevalence. Although consistent with some sequential diagnostic frameworks (Nordanstig et al., 2024), future studies should report PAD and AC both independently and jointly to better capture their overlap and combined risk profile (Lanzer et al., 2021; Criqui et al., 2021).

**References**

1. Nordanstig, J., Behrendt, C. A., Baumgartner, I., Belch, J., Bäck, M., Fitridge, R., et al. (2024). Editor’s Choice – European Society for Vascular Surgery (ESVS) 2024 Clinical Practice Guidelines on the Management of Asymptomatic Lower Limb Peripheral Arterial Disease and Intermittent Claudication. European Journal of Vascular and Endovascular Surgery, 67, 9–96. https://doi.org/10.1016/j.ejvs.2023.08.067
2. Criqui, M. H., Matsushita, K., Aboyans, V., Hess, C. N., Hicks, C. W., Kwan, T. W., McDermott, M. M., Misra, S., Ujueta, F., & Gornik, H. L. (2021). Lower Extremity Peripheral Artery Disease: Contemporary Epidemiology, Management Gaps, and Future Directions: A Scientific Statement From the American Heart Association. Circulation, 144(9), e171–e191. https://doi.org/10.1161/CIR.0000000000001005
3. Lanzer, P., Boehm, M., Sorribas, V., Thiriet, M., Janzen, J., Zeller, T., St Hilaire, C., & Shanahan, C. (2021). Medial vascular calcification revisited: Review and perspectives. European Heart Journal, 42(15), 1519–1531. https://doi.org/10.1093/eurheartj/ehaa564

Minor Comments:
Clarity and Presentation:

44. The abstract could be more concise. For example, the methodology section repeats details (e.g., ABI calculation methods) that are elaborated in the main text.

We appreciate the reviewer’s observation. In response, we have **condensed the abstract**, particularly the methodology section, to avoid redundancy with the main text. The revised abstract now briefly states that three ABI calculation methods (highest, lowest, and average systolic pressure) were used, without further procedural detail, as these are fully described in the Methods section. This change streamlines the abstract and allows more space to emphasize key findings and conclusions, in line with journal guidelines for concise abstracts.

45. Table 1 provides extensive demographic and clinical data, but some variables (e.g., education level, treated hypothyroidism) are not discussed in the results or discussion. Consider streamlining or highlighting the most relevant variables.

We thank the reviewer for the suggestion. Table 1 was streamlined by removing variables not discussed in the Results/Discussion or, when relevant (e.g., education), incorporated into the narrative for consistency.

Statistical Analysis:

46. The use of Poisson regression with robust variance is appropriate, but the manuscript should clarify why this method was chosen over logistic regression, especially given the cross-sectional design.

We appreciate the reviewer’s comment. We chose **Poisson regression with robust variance** instead of logistic regression because our main objective was to estimate **prevalence ratios (PRs)** rather than odds ratios (ORs). In cross-sectional studies, logistic regression often overestimates the magnitude of association when the outcome is common (prevalence >10%), as is the case for peripheral arterial disease (PAD) and arterial calcification (AC) in our population.

Poisson regression with robust variance is a well-recognized alternative that provides a direct and more interpretable estimate of PRs, avoiding the inflation of effect sizes inherent to ORs in common outcomes. This approach has been recommended in epidemiological literature for binary outcomes in cross-sectional designs (Barros & Hirakata, 2003; Zou, 2004).

**References**

- Barros AJ, Hirakata VN. Alternatives for logistic regression in cross-sectional studies: an empirical comparison of models that directly estimate the prevalence ratio. BMC Med Res Methodol. 2003;3:21. doi:10.1186/1471-2288-3-21
- Zou G. A modified Poisson regression approach to prospective studies with binary data. Am J Epidemiol. 2004;159(7):702-6. doi:10.1093/aje/kwh090

47. The power calculation is well-described, but it would be helpful to include the actual observed prevalences alongside the expected values for comparison.

We appreciate the reviewer’s observation. The observed prevalences for each ABI criterion were already included in the original version of the manuscript; however, we recognize that their presentation could be clearer. Following the reviewer’s suggestion, we have revised the phrasing in the Methods section to make the comparison between expected and observed prevalences more explicit. The updated text now reads:

We calculated the sample size power of 643 subjects to estimate the prevalence of peripheral arterial disease (PAD) for each criterion using the power command in STATA® V18. We employed the formula for estimating a proportion. We considered expected PAD prevalences based on Schröder's study of 3.7%, 5.4%, and 14.6% for the highest, average, and lowest ABI criteria, respectively. [28] For comparison, the corresponding prevalences observed in our study were 7.8%, 15.4%, and 28.2% By configuring a two-tailed analysis and a 95% confidence level, the statistical power achieved was 99%, 100%, and 100%, for the highest, average, and lowest ABI respectively.

Figures and Tables:

48. Figure 1 (diagnostic flowchart) is clear but could be simplified for readability (e.g., reduce redundant text).

We appreciate the reviewer’s suggestion. Figure 1 has been revised to improve readability by shortening sentences, removing redundant text, and presenting key steps more concisely without altering the diagnostic logic.

49. Tables 3 and 4 are dense and could benefit from reorganization (e.g., grouping related variables) to improve clarity.

We thank the reviewer for this valuable suggestion. In the revised version, Tables 3 and 4 have been reorganized to enhance clarity and readability. Variables are now grouped into logical categories (e.g., Demographics, Past Medical History, Clinical Evaluation, Laboratory Findings,), allowing related variables to be presented together.

References:

50. The references are comprehensive, but some citations (e.g., for ABI cut-off points) could be updated to reflect the most recent guidelines (e.g., 2024 ACC/AHA guidelines cited in the manuscript).

We thank the reviewer for this observation. In the revised manuscript, we have clarified the rationale for the ABI cut-off values and updated the references to reflect the most recent clinical guidelines.

For peripheral artery disease (PAD), we used the universally accepted diagnostic threshold of ABI ≤ 0.90, as recommended by all major clinical practice guidelines, including the 2024 ACC/AHA (Gornik et al., 2024) , the 2024 ESVS guidelines (Nordanstig et al., 2023), and recent AHA scientific statements (Criqui et al., 2021). This cut-off is supported by robust evidence linking it to ≥50% lower extremity arterial stenosis, with good sensitivity and specificity compared with angiograph. Values between 0.91–1.30 are considered normal.

For arterial calcification, there is no single universally accepted cut-off. Most studies and some guidelines use ABI > 1.30 to suspect medial arterial calcification (Mönckeberg sclerosis), particularly in patients with diabetes or chronic kidney disease (Adragão et al., 2012). Others employ ABI > 1.40 for higher specificity (Hoek et al., 2021). Our study applied the >1.30 threshold, as it is the most commonly reported in the literature, but we have added text acknowledging that >1.40 is also used in some consensus statements.

The revised section now explicitly cites the 2024 intersocietal IWGDF/ESVS/SVS guidelines on PAD in people with diabetes and foot ulcer (Fitridge et al., 2024), the 2024 ACC/AHA guideline on PAD management (Gornik et al., 2024), and other major guidelines, specifying the chosen cut-off values for both PAD and arterial calcification.

References

1. Criqui MH, Matsushita K, Aboyans V, et al. Lower Extremity Peripheral Artery Disease: Contemporary Epidemiology, Management Gaps, and Future Directions: A Scientific Statement From the American Heart Association. *Circulation*. 2021;144(9):e171-e191. doi:10.1161/CIR.0000000000001005.
2. Nordanstig J, Behrendt CA, Baumgartner I, et al. European Society for Vascular Surgery (ESVS) 2024 Clinical Practice Guidelines on the Management of Asymptomatic Lower Limb Peripheral Arterial Disease and Intermittent Claudication. *Eur J Vasc Endovasc Surg*. 2023. doi:10.1016/j.ejvs.2023.08.067.
3. Gornik HL, Aronow HD, Goodney PP, et al. 2024 ACC/AHA/AACVPR/APMA/ABC/SCAI/SVM/SVN/SVS/SIR/VESS Guideline for the Management of Lower Extremity Peripheral Artery Disease. *J Am Coll Cardiol*. 2024;83(24):2497-2604. doi:10.1016/j.jacc.2024.02.013.
4. Fitridge R, Chuter V, Mills J, et al. The intersocietal IWGDF, ESVS, SVS guidelines on peripheral artery disease in people with diabetes and a foot ulcer. *Diabetes Metab Res Rev*. 2024;40(5):e3686. doi:10.1002/dmrr.3686.
5. Adragão T, Pires A, Branco P, et al. Ankle–brachial index, vascular calcifications and mortality in dialysis patients. *Nephrol Dial Transplant*. 2012;27(1):318-325. doi:10.1093/ndt/gfr233.
6. Hoek AG, Zwakenberg SR, Elders PJ, et al. An elevated ankle-brachial index is not a valid proxy for peripheral medial arterial calcification. *Atherosclerosis*. 2021;323:13-19. doi:10.1016/j.atherosclerosis.2021.03.010.
